# Cyclodextrin Polymers as Delivery Systems for Targeted Anti-Cancer Chemotherapy

**DOI:** 10.3390/molecules26196046

**Published:** 2021-10-06

**Authors:** Noemi Bognanni, Maurizio Viale, Alessia Distefano, Rita Tosto, Nadia Bertola, Fabrizio Loiacono, Marco Ponassi, Domenico Spinelli, Giuseppe Pappalardo, Graziella Vecchio

**Affiliations:** 1 Dipartimento di Scienze Chimiche, Università degli Studi di Catania, Viale A. Doria 6, 95125 Catania, Italy; noemibognanni91@gmail.com (N.B.); alessiadistefano92@tiscali.it (A.D.); ttstt.rita@live.it (R.T.); 2 IRCCS Ospedale Policlinico San Martino, U.O.C. Bioterapie, L.go R. Benzi 10, 16132 Genova, Italy; nadia.bertola@gmail.com; 3 CNR Istituto di Cristallografia, Sede di Catania, Via Paolo Gaifami 18, 95126 Catania, Italy; giuseppe.pappalardo@cnr.it; 4 IRCCS Ospedale Policlinico San Martino, U.O.C. Immunologia, L.go R. Benzi 10, 16132 Genova, Italy; fabrizio.loiacono@hsanmartino.it; 5 IRCCS Ospedale Policlinico San Martino, U.O.S. Proteomica e Spettrometria di Massa, L.go R. Benzi 10, 16132 Genova, Italy; marco.ponassi@hsanmartino.it; 6 Dipartimento di Chimica “G. Ciamician”, Alma Mater Studiorum-University of Bologna, Via F. Selmi 2, 40126 Bologna, Italy; domenico.spinelli@unibo.it; 7 Consorzio Interuniversitario di Ricerca in Chimica dei Metalli nei Sistemi Biologici (CIRCMSB), 70121 Bari, Italy

**Keywords:** carbohydrates, cyclodextrins, nanoparticles, cancer, doxorubicin, oxaliplatin

## Abstract

In the few last years, nanosystems have emerged as a potential therapeutic approach to improve the efficacy and selectivity of many drugs. Cyclodextrins (CyDs) and their nanoparticles have been widely investigated as drug delivery systems. The covalent functionalization of CyD polymer nanoparticles with targeting molecules can improve the therapeutic potential of this family of nanosystems. In this study, we investigated cross-linked γ- and β-cyclodextrin polymers as carriers for doxorubicin (ox) and oxaliplatin (Oxa). We also functionalized γ-CyD polymer bearing COOH functionalities with arginine-glycine-aspartic or arginine moieties for targeting the integrin receptors of cancer cells. We tested the Dox and Oxa anti-proliferative activity in the presence of the precursor polymer with COOH functionalities and its derivatives in A549 (lung, carcinoma) and HepG2 (liver, carcinoma) cell lines. We found that CyD polymers can significantly improve the antiproliferative activity of Dox in HepG2 cell lines only, whereas the cytotoxic activity of Oxa resulted as enhanced in both cell lines. The peptide or amino acid functionalized CyD polymers, loaded with Dox, did not show any additional effect compared to the precursor polymer. Finally, studies of Dox uptake showed that the higher antiproliferative activity of complexes correlates with the higher accumulation of Dox inside the cells. The results show that CyD polymers could be used as carriers for repositioning classical anticancer drugs such as Dox or Oxa to increase their antitumor activity.

## 1. Introduction

In the few last years, nanosystems have emerged as a potential therapeutic approach to improve the efficacy and selectivity of drugs [1,2]. The prolonged blood circulation time of nanoparticles and passive targeting for EPR (enhanced permeability and retention) effect of the tumor on inflamed tissues attracted nanomedicine [3,4]. A variety of nanoparticles were investigated for their biomedical applications [5,6].

Cyclodextrins (CyDs) are cyclic oligosaccharides of α-1,4-linked d(+)-glucopyranose with the property to act as molecular containers [7,8,9]. They are widely used as excipients in pharmaceutical formulations and investigated as therapeutic cavities [10,11]. CyDs were also used to build nanoparticles (NPs) for their properties and biocompatibility. CyDs can form NPs by self-assembly [9,12], but these NPs often have low physical stability and low applicability as drug delivery systems [13]. Other families of CyD-based NPs were developed: hybrid NPs with an inorganic core [14], supramolecular systems and polymeric CyDs [15,16,17,18,19,20].

Polymeric CyD nanoparticles were synthesized through a variety of strategies [21,22,23]. Each polymer has several cavities in this class of systems and can bind more than a guest molecule with a higher affinity than the single cyclodextrin.

In many cases, epichlorohydrin (EPI) has been used as a suitable cross-linker agent to synthesize CyD polymers [24].

The properties of CyD based NPs were modulated with different approaches to improve their applicability and selectivity in biological systems. The most successful example is CALAA-01 as a gene delivery system. NPs with recognition units were made functional by exploiting adamantane residue affinity for the β-CyD cavity. Adamantane derivatives of transferrin were used as the target unit and successful results were obtained [25]. CyD based NPs were modified with receptor-specific targeting ligands (peptides, sugars, aptamers or vitamins) which could allow the delivery vehicle to undergo receptor-mediated endocytosis. We have decorated CyD cross-linked polymers with RGD moiety through its functionalization with adamantane [26].

Active targeting of the CyD polymers to selectively recognize cancer cells was obtained by covalent attachment of a variety of moieties, such as the luteinizing hormone-releasing hormone analog LHRHa (9 amino acids), folic acids, biotin [27,28,29,30,31,32]. CyD polymers have also been conjugated with other therapeutic small molecules, such as methylprednisolone [33] or diagnostic molecules such as DOTA [34].

**Figure 1:**
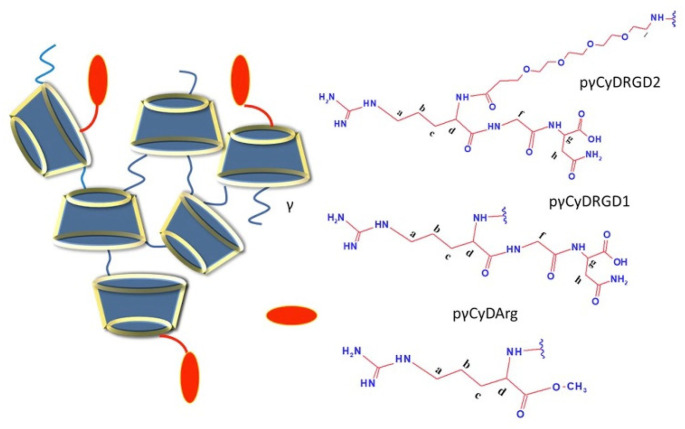
Structure of functionalized cross-linked γ-CyD polymers.

In this study, we investigated cross-linked CyD polymers as carriers for doxorubicin (Dox) and oxaliplatin (Oxa), knowing that CyDs can include Dox and Oxa [27,35,36,37,38]. Many authors reported that γ-CyD can include Dox better than β-CDs and increases its bioavailability [27,36,37,38]. Oxa can be included in both β and γ-CyD cavities with similar stability constants [35]. With the aim of binding to the avβ3, a5β1 and avβ6 integrin receptors, we also functionalized the γ-CyD polymer with COOH functionalities (pγCyDA) with arginine-glycine-aspartic (RGD) [39] or with Arg moieties (Figure 1). We studied the functionalized γ-CyD polymers as Dox carriers. Dox is an anthracycline drug very toxic, mainly cardiotoxic, and many formulations have been studied to reduce this toxicity [40].

We tested polymer/drug complexes in lung carcinoma A549 and hepatocarcinoma HepG2 cell lines in comparison with the cross-linked γ-CyD polymer (pγCyD) and anionic β-CyD polymer. A549 and HepG2 cell lines derived from neoplasms placed in the fourth leading causes of death worldwide [41,42].

## 2. Results and Discussion

### 2.1. Synthesis and Characterization

EPI cross-linked polymer pγCyDA functionalized with the tripeptide RGD directly or by a PEG4 chain (RGDPEG4) or with Arg methyl ester through a condensation reaction in DMF. The final products were characterized with NMR (Figures S1–S8).

In the ^1^H NMR spectra, signals are broad as typically found for similar systems (Figures S2–S4). The signals of CyD protons and RGD or Arg moiety can be easily assigned in the spectra.

The integrated intensities of the signals of CyD Hs-1 and RGD protons gave the number of RGD moieties in the polymer. As for pγCyDRGD1, this value suggested that about 80% of CyD units (about 21 CyD) were modified with RGD whereas in the case of pγCyDRGD2 only 50% of CyD units (about 14 CyD) were modified.

NOESY spectra of the pγCyDRGD2 showed NOE correlations between CyD and RGD protons (Figure S7). This is consistent with the conjugation of RGD and CyD. The ^13^C NMR spectra showed the alkyl carbons of the RGD moiety and the C=O and C=N carbons at 160–180 ppm (Figure S8). Similarly, ^1^H NMR spectra of pγCyDArg show the conjugation of Arg to pγCyDA.

The integrated intensities of the Hs-1 signals of CyD and the broad signal at 1.86–1.40 ppm provided the number of Arg moieties in the polymer. This value suggested that about 31% of CyD units were functionalized with Arg, which corresponds to about 9 units of Arg in the polymer.

### 2.2. Solubility Experiments

**Figure 2:**
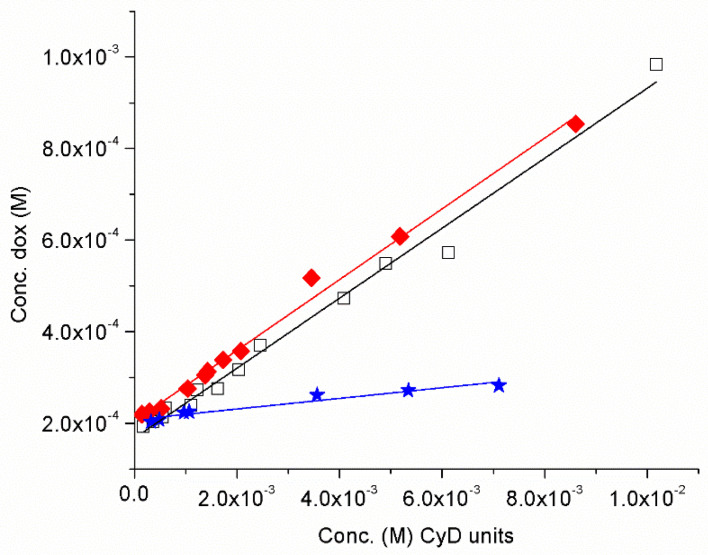
Dox solubility (pH 7.4) versus the amount of pγCyDA (ν), pβCyDA (□) and pγCyD (★) (reported as CyD cavity concentration).

Oxa has the water solubility of 4 mg/mL (10^−2^ M) and we investigated only the solubility of Dox (water solubility of free Dox 7.40 × 10^−5^ M pH 7.4) in the presence of pγCyDA, pβCyDA and pγCyD at pH 7.4. A typical experiment is reported in Figure S9. We carried out solubility experiments to investigate the role of COOH functional groups in the polymers in the interaction between the polymers and Dox, a cationic drug at physiological pH. We investigated the systems by a phase solubility analysis [8]. The solubility phase diagrams were obtained by plotting the Dox concentration versus the polymer concentration (Figure 2).

In particular, we reported the Dox solubility versus the CyD unit concentration for a better comparison among the different polymers [43,44]. The phase diagrams are A_L_-type and have a slope < 1. Complexation efficiency (CE) values of CyD cavity are 0.083 for pγCyDA, 0.082 for pβCyDA and 0.011 pγCyD, suggesting that the presence of carboxylate groups improves the interaction with Dox. While pγCyD only slightly improves the solubility of Dox, the other polymers increase the solubility of Dox very significantly, suggesting a higher affinity for the guest. We did not find a better affinity of pγCyDA compared to pβCyDA. The polymers are EPI cross-linked polymers and an influential role of the network in the Dox interaction can also be proposed.

### 2.3. Antiproliferative Activity

**Figure 3:**
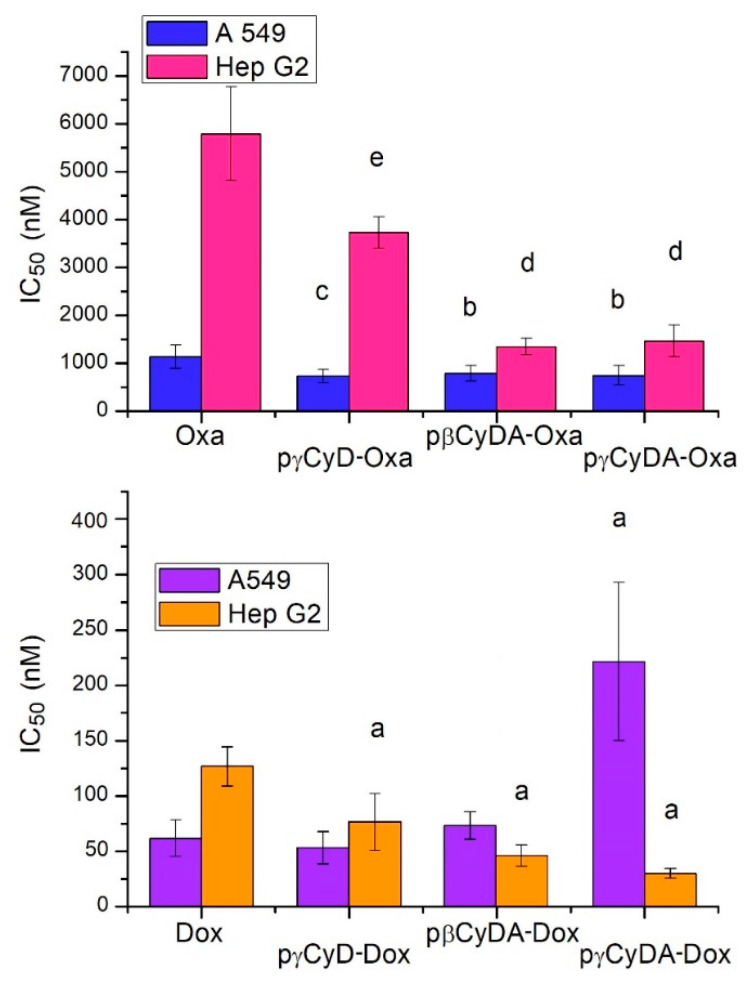
IC_50_ (nM) of CyD polymer-Dox/Oxa in human A549 and HepG2 tumor cells. ^a^ *p* < 0.001, vs. Dox; ^b^ *p* < 0.05 vs. Oxa; ^c^ *p* < 0.02 vs. Oxa; ^d^ *p* < 0.001 vs. Oxa; ^e^ *p* < 0.01 vs. Oxa.

Cell proliferation assay was performed with Dox in the presence of pγCyDA, pβCyDA, pγCyD, pγCyDRGD1, pγCyDRGD2, pγCyDArg, compared to free Dox. At the same time, antiproliferative activity for Oxa was performed only in the presence of pγCyDA, pβCyDA and pγCyD and compared to free Oxa (Figure 3).

All polymers alone did not show any toxicity at µM concentration.

In A549 cells, Dox-polymer complexes showed a similar or even significantly lower antiproliferative activity (pγCyDA-Dox) in comparison to free Dox.

Conversely, in HepG2 cells pγCyDA-Dox, pβCyDA-Dox and pgCyD-Dox showed always a noteworthy and significant decrease of IC_50_ value as compared to Dox alone.

When Oxa was administered to A549 and HepG2 cells in the presence of pγCyDA, pβCyDA and pγCyD its antiproliferative activity was always significantly improved as compared to the treatment with Oxa alone (Figure 3).

**Figure 4:**
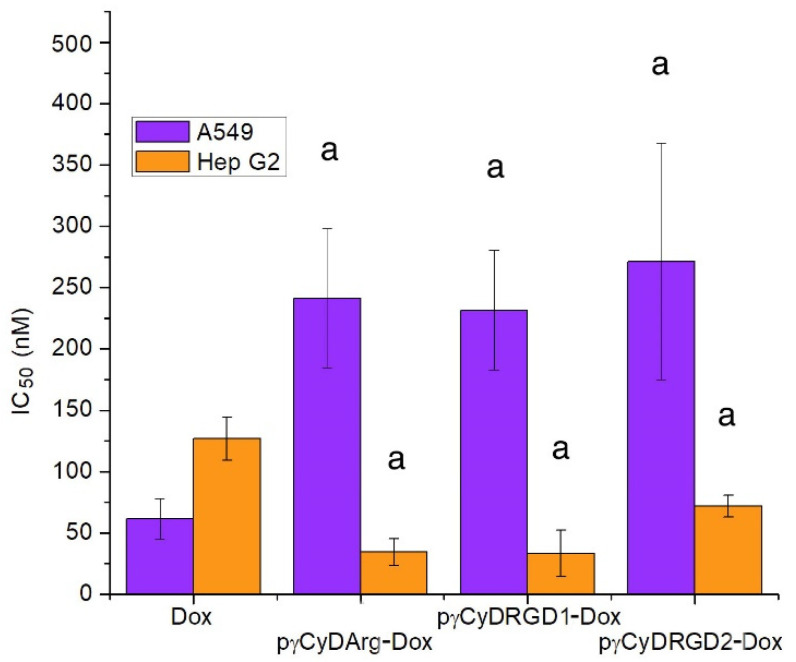
IC_50_ (nM) of CyD functionalized polymer-Dox in human A549 and HepG2 tumor cells. ^a^ *p* < 0.001, vs. Dox.

In order to study the possible effect of functionalization of cross-linked polymers, we synthesized RGD and Arg functionalized polymers and tested the new complexes pγCyDRGD1-Dox, pγCyDARGD2-Dox, and pγCyDArg-Dox (Figure 4).

These RGD functionalized polymers showed an antiproliferative activity similar to that found for the Dox complex with pγCyDA (Figure 4), both in A549 and in HepG2 cells. This suggests that Dox activity is independent of the functionalization with RGD or Arg. The reason for the limited effect of RGD units can be due to the low availability of the targeting moieties.

In particular, the fact that pγCyDRGD2-Dox showed a significantly lower antiproliferative effect than free Dox may be due to the presence of PEG chain that can modify the features of the system.

This result is of interest because the effect of the polymers on the Dox toxicity is generally significant, while in many cases reported elsewhere the presence of NPs reduced the cytotoxicity of the drug. The entrapment into NPs may reduce the ability of the drug to cross the cell membrane. In this case, we found that anionic polymers can include Dox better than the pCyD. This data can explain the decreased activity in A549 cells. On the contrary, in the case of HepG2 cells, pγCyDA is the most effective system. Probably the type of transporters of the HepG2 cells may explain this behavior [45].

### 2.4. Dox Intracellular Accumulation

We cytofluorimetrically evaluated the drug uptake in A549 and HepG2 cells after exposure to equal concentrations of Dox/CyD complexes and Dox alone to prove that the difference of anti-proliferative activity of Dox/CyD polymers compared to free Dox was determined by a differential Dox accumulation into target cells.

Our results clearly show that Dox accumulation was strictly dependent on the type of CyD-polymer utilized for drug delivery although with evident differences between A549 and HepG2 cells (Figures S10 and S11).

**Figure 5:**
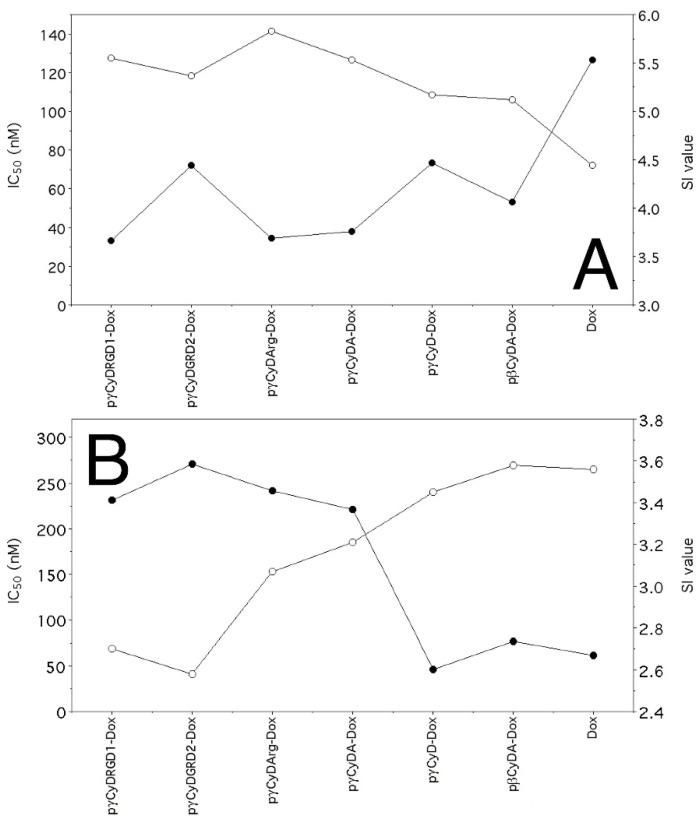
Correlation between IC_50_s (●) and staining index (SI, ◯) values calculated as described in Materials and Methods. (**A**), HepG2 cells; (**B**), A549 cells.

In fact, the Dox uptake in HepG2 cells in the presence of the CyD polymers was always significantly greater than the accumulation of Dox alone (*p* < 0.05). These data are inversely correlated with the analysis of the antiproliferative activity of such complexes (r = −0.924, *p* = 0.0144), which always had IC_50_ values lower than that of the parent compound alone (Figure 5).

On the contrary, in A549 cells, where Dox-pCyD complexes always had lower antiproliferative activity than Dox, the accumulation of this drug was mainly lower for Dox/CyD polymers than for the parent compound (*p* < 0.05 except for pγCyDA-Dox with a *p* < 0.10; correlation: r = −0.883 *p* = <0.01; Figure 5).

Altogether, these data demonstrate that the higher antiproliferative activity of our complexes correlates with the higher ability of CyD complexes to target and accumulate Dox into the cells.

## 3. Conclusions

Anionic cross-linked β and γCyD polymers and some of their conjugates with RGD or Arg were investigated. RGD and Arg were linked to the γCyD polymer with the aim of targeting tumor cells. The polymers formed stable nanoparticles of 20–30 nm.

In the literature, there are many studies on Dox nanoformulation based on CyDs and few studies on Oxa [7,27]. Some formulations are based on self-aggregates exploiting triggering behavior but, in many cases, critical stability [7,27,46,47].

We found that the cross-linked γCyD polymer can include the platinum anticancer drug Oxa and the topoisomerase inhibitor Dox, in this case improving its solubility in water at a physiological pH. The carboxylic groups in the anionic polymers may cooperate for the interaction with the amine Dox.

We tested the cross-linked polymers as nanocarriers of Dox and Oxa. We choose A549 and HepG2 cell lines as the target for our experiments due to their origin from tumor histotypes that represent two of the first leading causes of death worldwide. Furthermore, both these tumor histotypes still represent a great challenge for chemotherapy despite the new therapeutic innovations represented by the application of molecular target-based anticancer drugs.

The antiproliferative activity data show that the complexation with the γ and βCyD polymers significantly improves Dox activity only against HepG2 cell line whereas the complexation with Oxa significantly improves its antitumor activity in both cell lines. In particular, the pharmacological effect of Dox depends on the tumor cell line and is not improved in the systems functionalized with RGD or Arg. A possible reason for the unsuccessful effect of RGD functionalized polymers can be due to the low availability of the moieties entrapped into the polymer network.

Most importantly, studies aimed at verifying Dox accumulation into target cells showed that the observed drug uptake strongly correlates with the antiproliferative activity of Dox/polymers in both A549 and HepG2 cells, thus demonstrating that the improvement of antiproliferative activity, at least in the case of Dox-polymer complexes, was basically linked to the differential uptake of the anticancer drug determined by the polymers.

On the basis of the literature [37,48] we can hypothesize that the functional groups of the anionic nanocarriers studied may interact with the surface receptors/transporters by affecting the drug uptake.

Although the reason of selectivity is not well clarified, the most important result is the increase of the antiproliferative activity of Dox and Oxa in the presence of CyD polymers as compared to the free drugs. This result is quite remarkable since the encapsulation of the drug in nanoparticles often produces a decrease in cytotoxicity in vitro compared to the free drug [7,27,46] and only in vivo experiments can show the advantage of the nanoformulations [7,37,46].

On the basis of our results, the CyD polymers here described deserve further studies to understand their different selectivity for tumor cell lines of different histological origin and the possibility to use these carriers for repositioning other classical anticancer drugs still used for cancer chemotherapy to improve their antitumor activity.

## 4. Materials and Methods

### 4.1. Reagents

Commercially available reagents were used directly unless otherwise noted. Soluble cross-linked γCyD polymer (pγCyD, 101 kDa, 54 CyD cavities), anionic γCyD polymer (pγCyDA, 54 kDa, 28 CyD cavities, average number of carboxymethyl group for cavity is 3) were purchased from Cyclolab. 1-(3-Dimethylaminopropyl)-3-ethyl-carbodiimide hydrochloride (EDC, TCI Tokyo Chemical Industries), N-Hydroxy succinimide (NHS, ALPHA AESAR); doxorubicin hydrochloride (Carbosynth), oxaliplatin (TCI Tokyo Chemical Industries), arginine methyl ester dihydrochloride (ArgOCH3, TCI Tokyo Chemical Industries) were used. Free base ArgOCH3 was obtained using a DEAE (OH- form) anionic column.

Sephadex G-15 and sephadex-DEAE A-25 were used for column chromatography.

Thin-layer chromatography (TLC) was carried out on silica gel plates. Carbohydrate derivatives were detected on TLC by UV, anisaldehyde test or ninhydrin.

### 4.2. NMR Spectroscopy

^1^H NMR spectra were recorded at 25 °C with a Varian UNITY PLUS-500 spectrometer at 499.9 and 125.7 MHz respectively, using standard pulse programs from the Varian library. Two-dimensional experiments (COSY, TOCSY, HSQC, and NOESY) were performed using 1K data points, 256 increments. The mixing times for NOESY were 200–400 ms. Spectra were referred to the solvent signal.

### 4.3. UV-Vis Spectroscopy

UV-Vis spectra were recorded with VersaWave microvolume UV/Vis spectrophotometer.

### 4.4. Dynamic Light Scattering

Dynamic light scattering (DLS) measurements were performed at 25 °C with Zetasizer Nano ZS (Malvern Instruments, UK) operating at 633 nm (He–Ne laser). The mean hydrodynamic diameter (d) of the NPs was calculated from intensity measurement after averaging the five measurements. The samples (1 mg/mL) were diluted in phosphate buffer (pH = 7.4) prepared in ultrapure filtered water (0.2 µm filter). The Z average values of commercial polymers were also measured: pγCyD 35 ± 3 nm, pγCDA 25 ± 2 nm, pβCDA 53 ± 5 nm.

### 4.5. Solid-Phase Synthesis of RGD-PEG4-

The RGD and PEG4RGD peptide were carried out on a Novabiochem TGR resin (0.25 mmol/g 0.1 mmol scale) using the Fmoc chemistry method. All Fmoc amino acids were introduced according to the Dic/oxyma activation method [49]. The 15-(9-fluorenylmethyloxycarbonyl)amino-4,7,10,13-tetraoxa-pentadecanoic acid (PEG4) moiety was linked to the peptidyl resin using a standard double coupling instrumental protocol. The following instrumental conditions were used for each coupling cycle: microwave power 220 W, reaction temperature 65 °C, coupling time 30 s and after 25 W, 90 °C, 90 s. The instrumental conditions used for the deprotection cycle were: microwave power 220 W, reaction temperature 70 °C, deprotection time 30 s and after 25 W, 75 °C, 30 s. The removal of Fmoc protection during synthesis was achieved using 20% piperidine solution in DMF. The peptides were cleaved off from the resin using a mixture of TFA/H2O/TIS (95:2.5:2.5 *v*/*v*/*v*). Crude peptides were recovered by precipitation with freshly distilled diethyl ether.

RGD: ^1^H NMR (D_2_O, 600 MHz) δ ppm): 4.00–3.96 (m, 1H, g); 4.00–3.96 (m, 2H, d); 3.58 (s, 2H, f); 3.16–3.10 (m, 2H, a); 2.71 (dd, *J* = 15.8, 6.8 Hz, 2H h); 1.84 (d, *J* = 7.3 Hz, 2H, c), 1.64–1.52 (m, 2H, b).

RGD-PEG4: ^1^H NMR (D_2_O, 600 MHz) δ (ppm): 4.54–4.48 (m, ^1^H, g); 4.22–4.16 (m, ^1^H, d); 3.81 (s, 1H, f); 3.69–3.46 (m, ^1^H, PEG4); 3.07 (t, *J* = 5.1 Hz, 2H, a); δ 2.72–2.58 (m, 2H, h); 2.50–2.40 (m, 2H, l PEG4); 1.79–1.45 (m, 4H, c and b).

### 4.6. Synthesis of pγCyDRGD1

pγCyDA (226 mg) was dissolved in DMF (10 mL). NHS (22.7 mg) and EDC (13.7 mg), previously dissolved in 500 μL of DMF, were added. A DMF solution of RGD (0.030 mg, 500 μL) was added drop by drop to the solution of pγCyDA during 12 h. The reaction mixture was stirred for 3 days at room temperature. TLC showed the formation of the new products. The product was isolated with a Sephadex G-15 column, using water as eluent.

The appropriate fractions were collected and analyzed by NMR. Acetone was added to the solid products and the precipitate was collected by centrifugation.

^1^H NMR: (D_2_O, 600 MHz) δ (ppm): 1.92–1.51 (m, c and b RGD); 2.64–2.55 (m, h RGD); 3.21–2.99 (m, a RGD); 4.16–3.18 (m, -3, -4, -5, -6 of CyD); 5.70–4.93 (m, H-1 of γ-CyD).

Dimension (DLS, Zaverage): 27 ± 3 nm.

### 4.7. Synthesis of pγCyDRGD2

The synthesis was carried out as reported for pγCyDRGD1, using RGD-PEG4.

After purification with Sephadex G-15, three fractions were collected and analyzed by NMR. The fractions were purified again with an anionic DEAE column chromatography.

The sample was precipitated by acetone, centrifuged and dried.

^1^H NMR: (D_2_O, 600 MHz) δ (ppm): 1.86–1.51 (m, c and b RGD); 2.6–2.55 (m, h RGD); 2.55–2.45 (m, l PEG4); 3.16–3.10 (t, a RGD); 4.10–3.19 (m, -3, -4, -5, -6 of CyD and H PEG4); 4.26 (t, *J* = 6.1 Hz, d RGD); 4.51–4.46 (m, g RGD); 5.75–4.83 (m, H-1, γ-CyD).

^13^C NMR (125 MHz, D_2_O) δ (ppm): 24.5 (b RGD); δ 24.9 (c RGD); 35.8 (h RGD); 40.7 (a RGD); 42.6 (f RGD); 60.5 (PEG4), 62.8 (PEG4); (C-3,-4,-5,-6, PEG); 79–81 (C4, γ-CyD); 102–104 (C1, γ-CyD); 156.7 (C=N, γ-CyD); δ 171.0 (COOH, γ-CyD); 174.5 (CONH); 176.2 177.4–177.8 (CONH); 178.0 (CONH2).

Dimension (DLS, Zaverage): 30 ± 3 nm.

### 4.8. Synthesis of pγCyDArg

pγCyDA (82.60 mg) was dissolved in 5 mL of DMF and NHS (5.86 mg in 500 μL) and EDC (9.82 mg in 500 μL) were added. Arginine methyl ester hydrochloride (ArgCOOCH_3_) was added under stirring. After 4 days the solution was purified via a Sephadex G-15 column and fractions obtained were analyzed through TLC chromatography.

The fractions positive to the anisaldehyde test were evaporated and characterized via NMR analysis. The product was precipitated with acetone and centrifuged.

^1^H NMR: (D_2_O, 600 MHz) δ 5.55–4.85 (m, H-1, γ-CyD); 4.31–3.12 (m, H-2, -3, -4, -5, -6 of CyD); 3.10 (m, a Arg); 1.86–1.4 (m, c and b Arg).

Dimension (DLS, Zaverage): 26 ± 3 nm.

### 4.9. Experiments of Solubility

Dox hydrochloride (50 µL, 0.017 M, water solution) was added to 0.200 mL of 8 different concentration solutions of the CyD polymers in phosphate buffer (50 mM, pH 7.4) as reported elsewhere [32]. The suspensions formed due to the Dox precipitation were sonicated for 10 min and incubated at 25 °C in the dark. After 12 h, suspensions were centrifuged at 10,800 RPM for 10 min at 25 °C. Dox concentrations of the samples were determined in the supernatant with UV/Vis spectroscopy at 482 nm (molar absorptivity of Dox 10.41 (mol^−1^ L cm^−1^). The CE (complexation efficient) was calculated from the slope of the straight line obtained. CE = Slope/(1 − Slope).

### 4.10. Antiproliferative Activity

Human cell lines A549 (lung, carcinoma) and HepG2 (liver, carcinoma) were plated 180 µL into flat-bottomed 96-well microtiter plates at 1200/mL and 2000/mL, respectively. After 6–8 h, cells were treated with 20 µL containing five concentrations of Dox or Oxa alone or loaded in CyD polymers diluted in normal saline solution. Plates were then processed as described elsewhere [50]. Dox with CyD polymers molar ratio (polymer/Dox) 1:15 were assayed. Oxa complexes had a molar ration polymer/Oxa of 1:30.

The concentrations inhibiting 50% cell growth (IC_50_) were calculated based on the analysis of the concentration–response curves. Each experiment was repeated 4–11 times.

### 4.11. Evaluation of Intracellular Accumulation of Dox Carried by CyD Complexes

A549 and HepG2 cells were plated in 6-well plates at 2.0 and 2.5 × 10^4^ cells/well in 3 mL, respectively. After 16 h, cells were treated with 1.2 μM Dox or Dox/CyD complexes. After 2 h cells were detached by exposure to trypsin-EDTA at 37 °C for 5 min, washed quickly once with cold PBS, and fixed with 0.3 mL of 3.7% para-formaldehyde in PBS containing 2% sucrose.

Untreated cells were assayed as well. The intracellular median fluorescence intensity of Dox was determined by flow cytometry (MACSQuant Analyzer 8, Miltenyi Biotec, Bologna, Italy) using 488 nm excitation and 655–730 nm emission filters. Data were analyzed with FowJo 10.7.2 software (Becton Dickinson, Milano, Italy)

Values were normalized using the Staining Index (SI):

$$\frac{\left[\mathrm{Mean}/\mathrm{Median}\;\mathrm{treated}\right]-\left[\mathrm{Mean}/\mathrm{Median}\;\mathrm{control}\right]}{2\times \mathrm{SD}\;\mathrm{control}}$$

### 4.12. Statistical Analysis of Data

The Mann–Whitney and the Wilcoxon signed-rank tests and the Pearson correlation coefficient were used for the statistical analysis of data.

## Data Availability

Data is contained within the article or Supplementary Materials.

## Supplementary Materials

The following are available online, NMR spectra of the new compounds, UV-Vis spectra of Dox/polymers, DLS of pCyD-polymers, dox accumulation experiments in HepG2 and A492 cells.

## Sample Availability

Not available.
