# Supplementary material for: Cyclodextrin Polymers as Delivery Systems for Targeted Anti-Cancer Chemotherapy
Source: Molecules. 2021 Oct 6;26(19):6046. doi: 10.3390/molecules26196046 (PMC8512365; doi:10.3390/molecules26196046)
Supplement: Supplementary file 1 [file molecules-26-06046-s001.zip › molecules-1322654-supplementary.pdf]

Supplementary Material

Cyclodextrin polymers as delivery systems for targeted anti-cancer chemotherapy

Noemi Bognanni,[1] Maurizio Viale,[2,*] Alessia Distefano,[1] Rita Tosto,[1,3] Nadia Bertola,[2] Fabrizio Loiacono[5], Marco Ponassi,[6] Domenico Spinelli,[7] Giuseppe Pappalardo,[3] and Graziella Vecchio[1,8*]

1 Dipartimento di Scienze Chimiche, Università degli Studi di Catania, Viale A. Doria 6, 95125 Catania, Italy; noemibognanni91@gmail.com, lessiadistefano92@tiscali.it

2 IRCCS Ospedale Policlinico San Martino, U.O.C. Bioterapie, L.go R. Benzi 10, 16132 Genova, Italy; mauri-zio.viale@hsanmartino.it, nadia.bertola@gmail.com

3 CNR Istituto di Cristallografia, Sede di Catania, Via Paolo Gaifami 18, 95126 Catania , Italy; ttstt.rita@live.it, giuseppe.pappalardo@cnr.it

5 IRCCS Ospedale Policlinico San Martino, U.O.C. Immunologia, L.go R. Benzi 10, 16132 Genova, Italy; fabri-zio.loiacono@hsanmartino.it

6 IRCCS Ospedale Policlinico San Martino, U.O.S Proteomica e Spettrometria di Massa, L.go R. Benzi 10, 16132 Genova, Italy; marco.ponassi@hsanmartino.it

7 Dipartimento di Chimica "G. Ciamician", Alma Mater Studiorum-University of Bologna, via F. Selmi 2, 40126 Bologna, Italy; domenico.spinelli@unibo.it

8 Consorzio Interuniversitario di Ricerca in Chimica dei Metalli nei Sistemi Biologici (CIRCMSB), Italy

* Correspondence: maurizio.viale@hsanmartino.it; gr.vecchio@unict.it

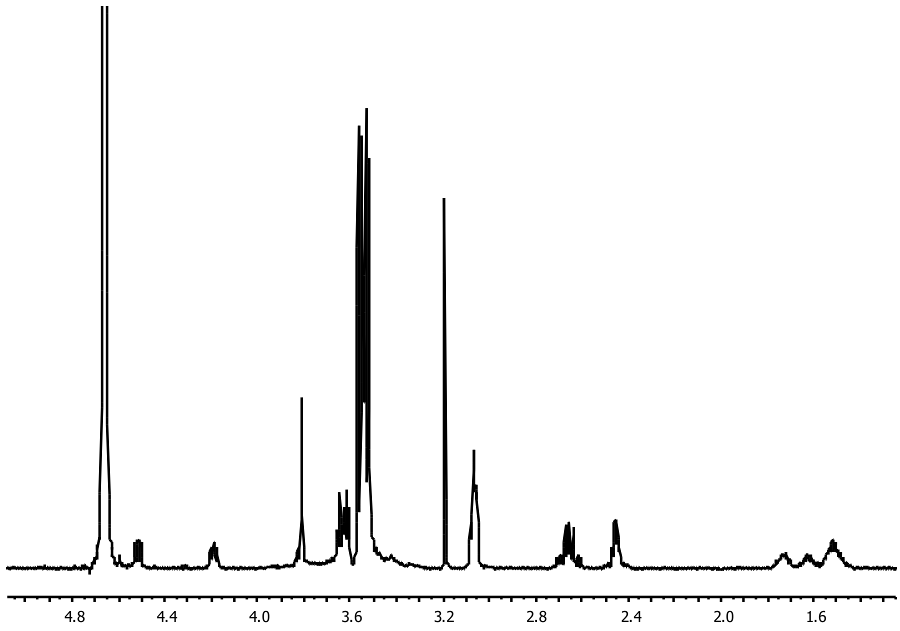

Figure S1. ^{1}H NMR spectrum of RGD-PEG4 (D_2O, 500 MHz).

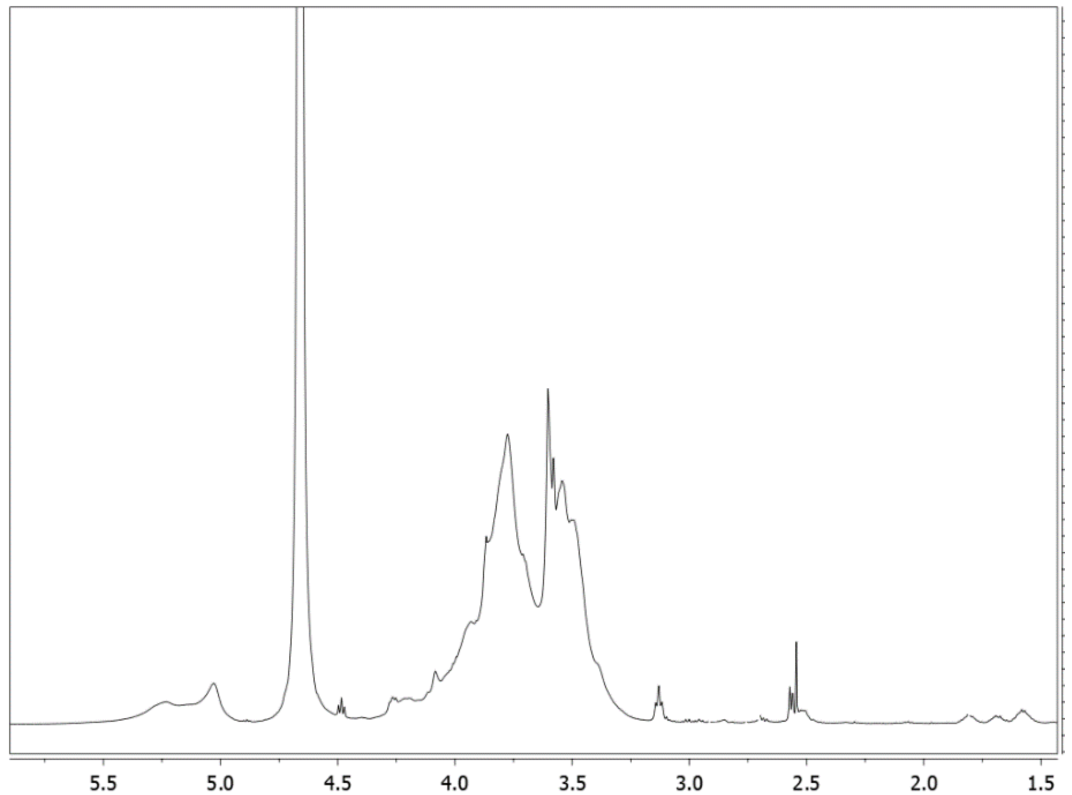

Figure S2. ^{1}H NMR spectrum of pγCyDARGD2 (D_2O, 500 MHz).

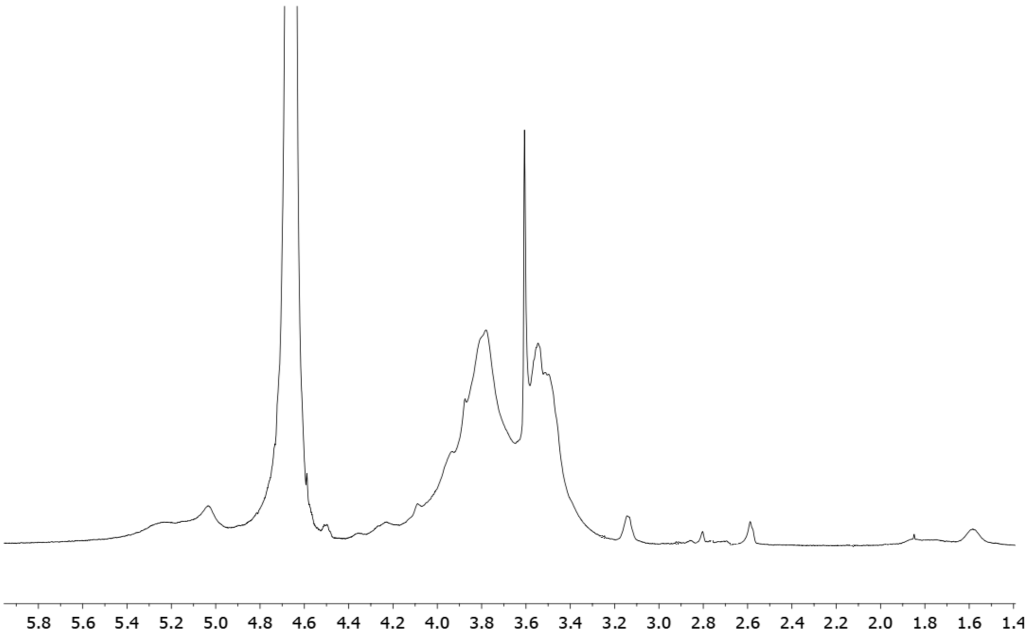

Figure S3. ^{1}H NMR spectrum of pγCyDARGD1 (D_2O, 500 MHz).

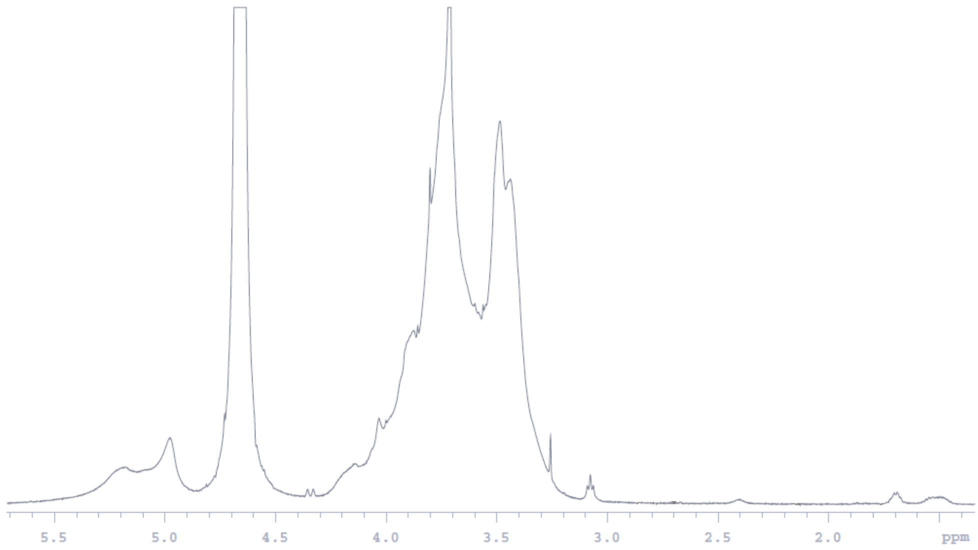

Figure S4. ^{1}H NMR spectrum of pγCyDARG (D_2O, 500 MHz).

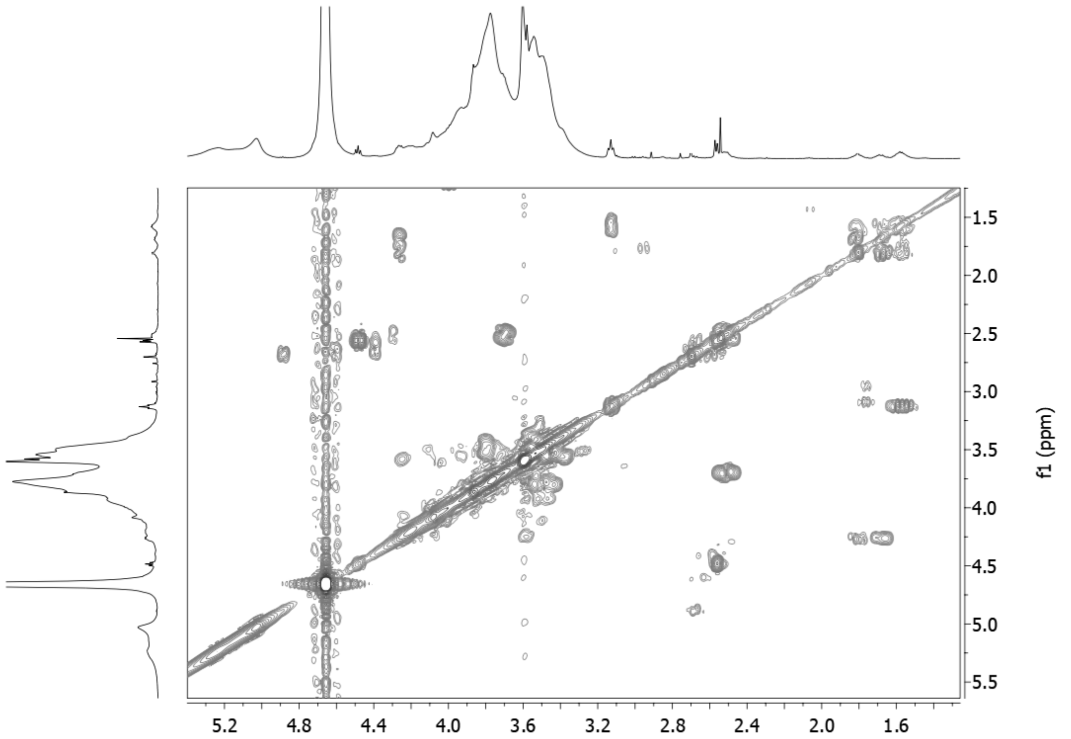

Figure S5. ^{1}H NMR COSY spectrum of pγCyDARGD2 (D_2O, 500 MHz).

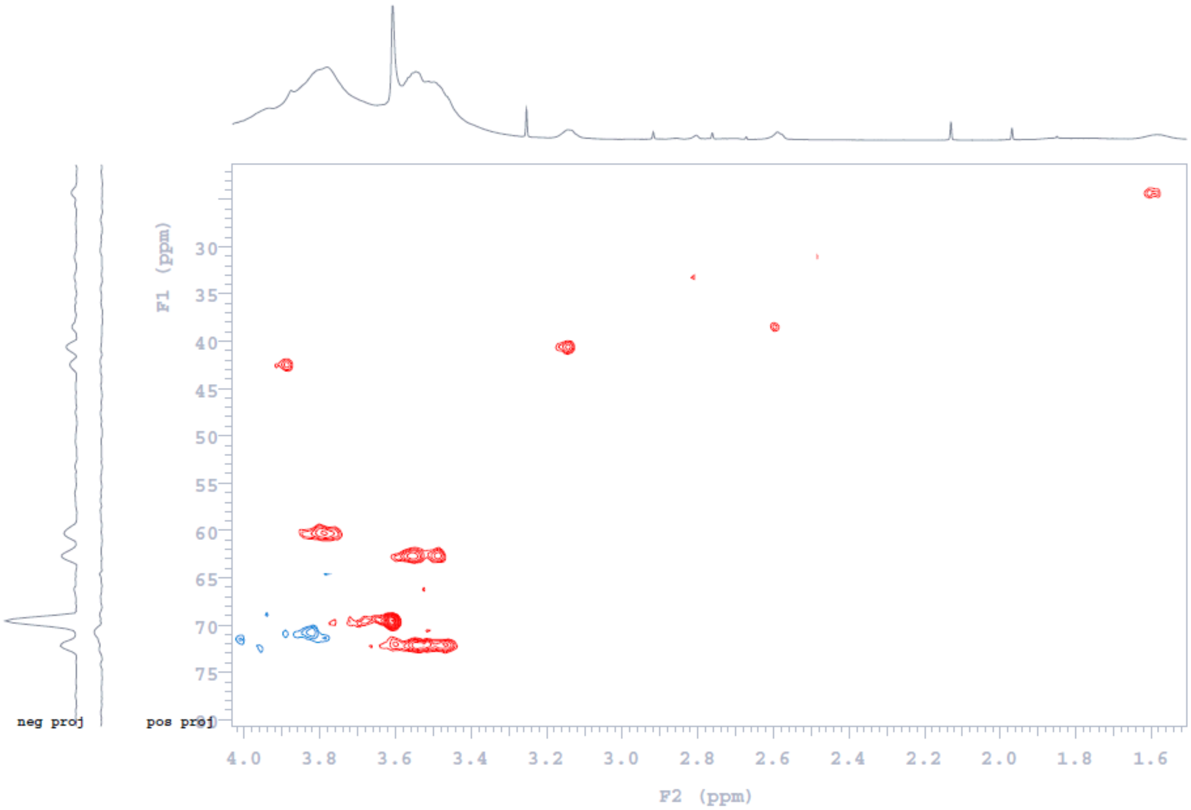

Figure S6. ^{1}H NMR HSQC spectrum of pyCyDARGD1 (D_2O, 500 MHz).

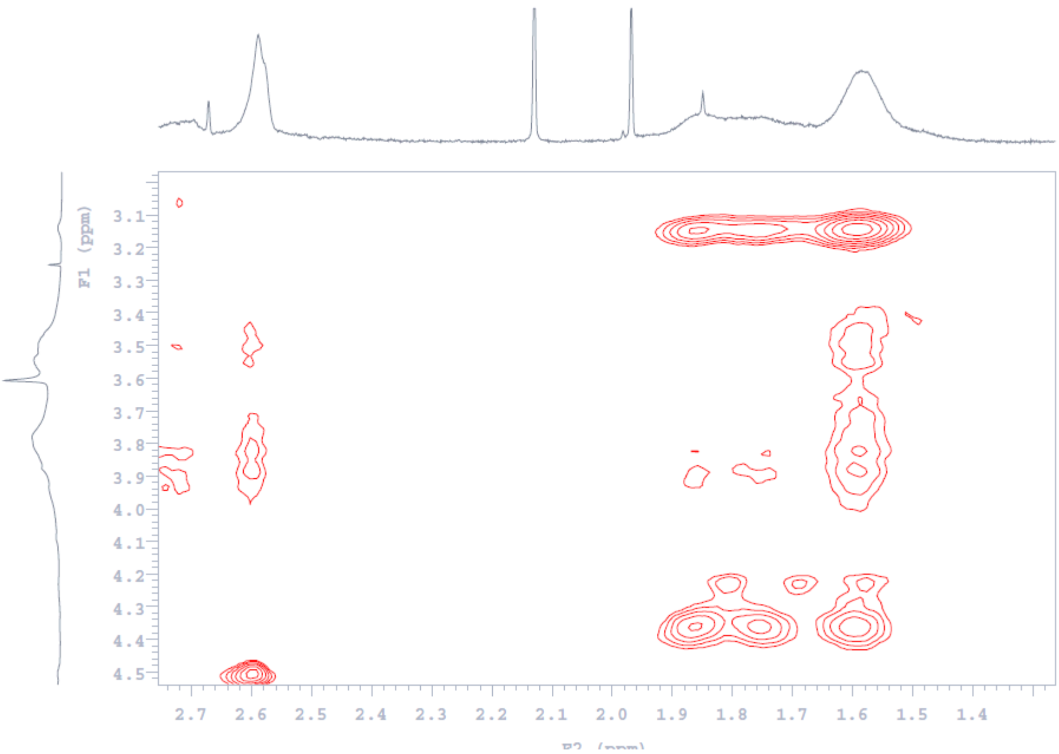

Figure S7. ^{1}H NMR NOESY spectrum of pyγCyDARGD2 (D_2O, 500 MHz).

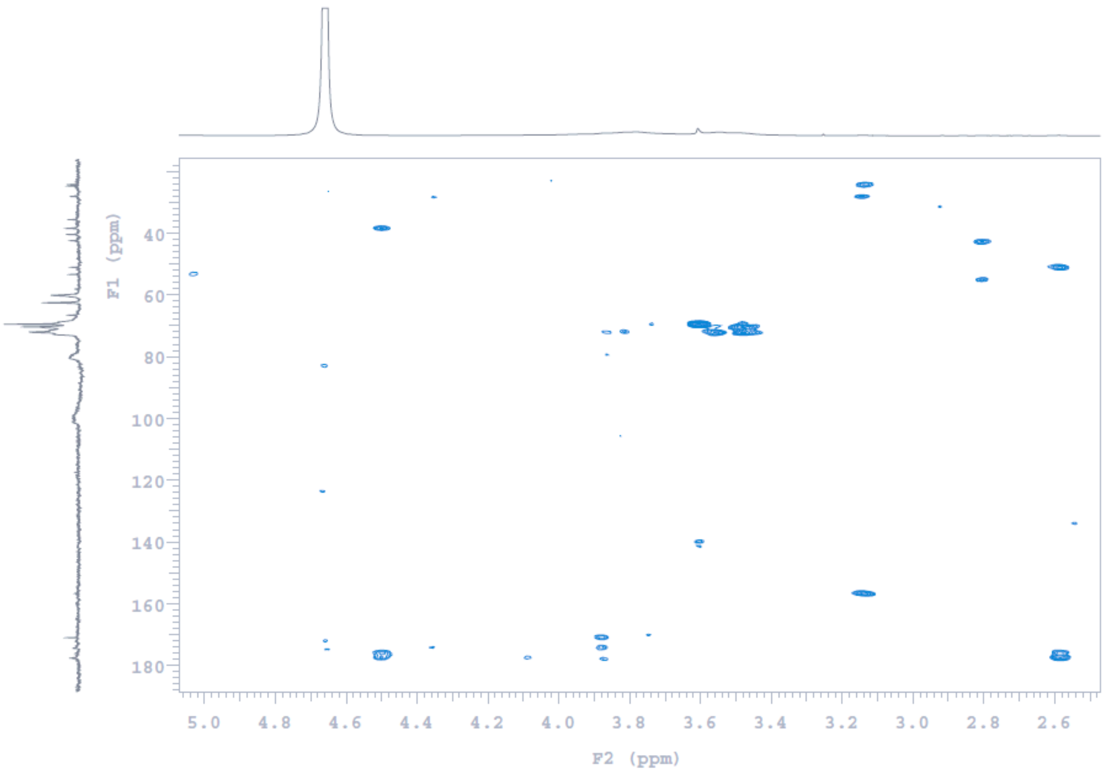

Figure S8. ^{1}H NMR HMBC spectrum of pγCyDARGD2 (D2O, 500 MHz).

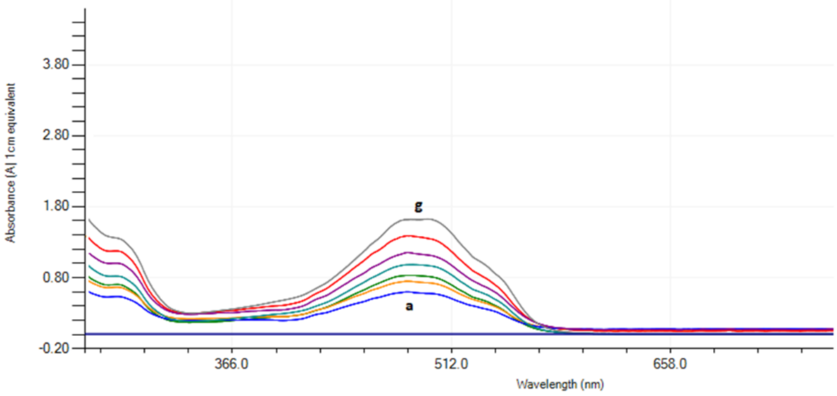

Figure S9. UV-Vis spectra of Dox-pγCyDA (from a to g: free Dox to Dox with 8 mg/mL of pγCyDA).

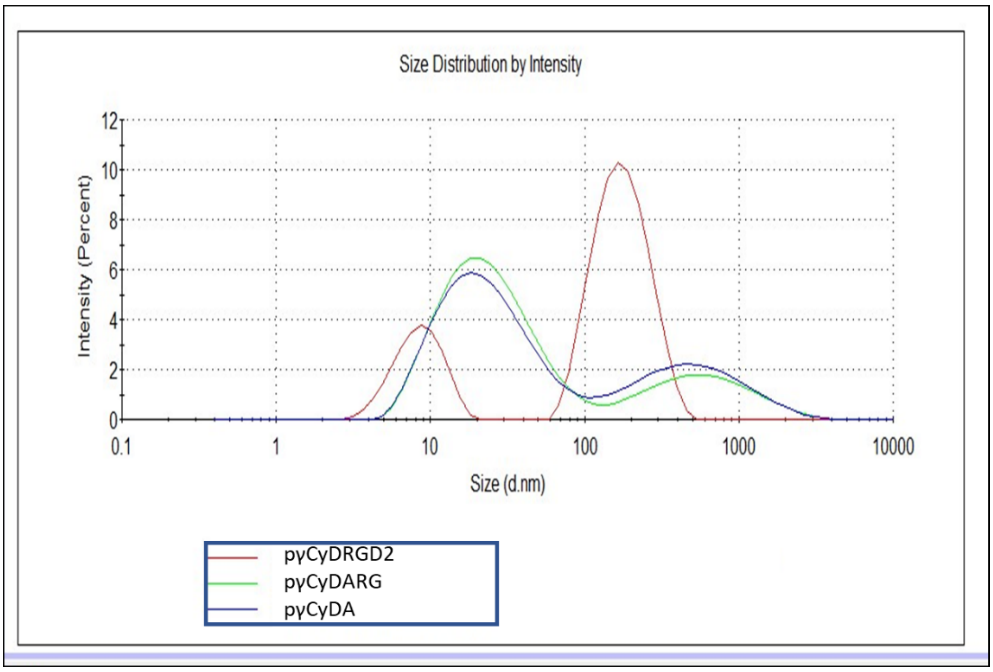

Figure S10. Size distribution by intensity (DLS) of pγCyD polymers in Phosphate Buffer (pH 7.4).

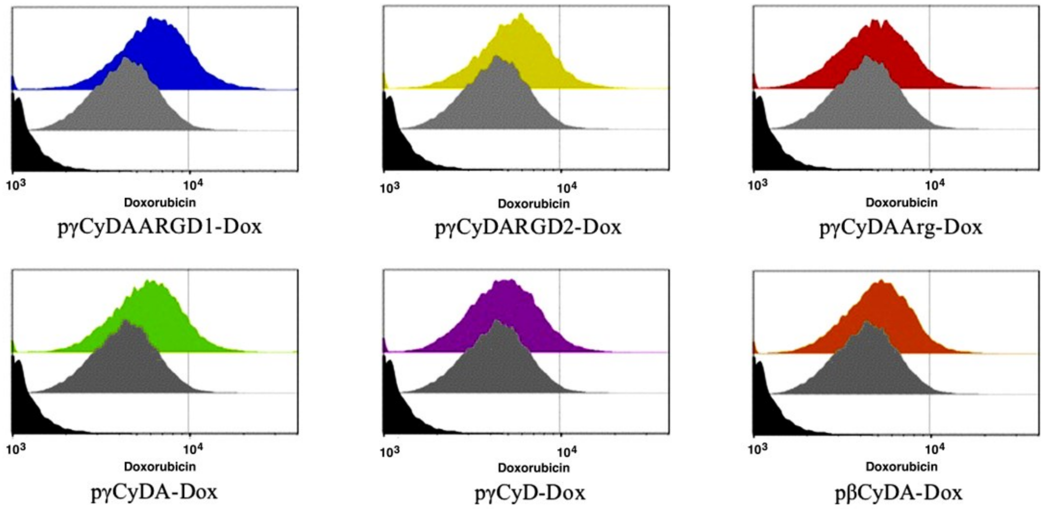

Figure S11. Representative experiment of Doxorubicin accumulation in HepG2 cells. Black histograms, control; dark gray histograms, Doxorubicin; colored histograms, as indicated below each box.

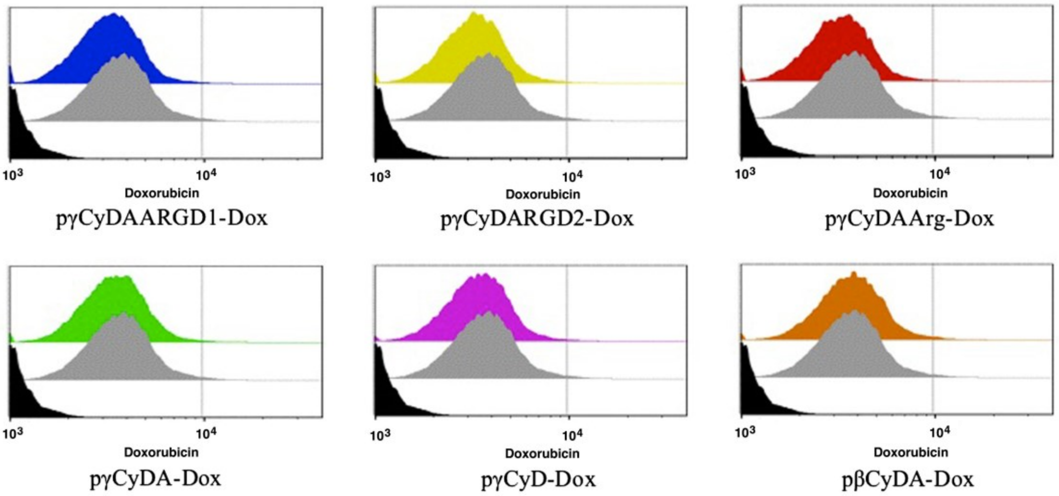

Figure S12. Representative experiment of Doxorubicin accumulation in A5492 cells. Black histograms, control; dark gray histograms, Doxorubicin; colored histograms, as indicated below each box.
